# Blue Light Improves Photosynthetic Performance and Biomass Partitioning toward Harvestable Organs in Saffron (*Crocus sativus* L.)

**DOI:** 10.3390/cells10081994

**Published:** 2021-08-05

**Authors:** Shirin Moradi, Mohsen Kafi, Sasan Aliniaeifard, Seyed Alireza Salami, Majid Shokrpour, Carsten Pedersen, Moein Moosavi-Nezhad, Jacek Wróbel, Hazem M. Kalaji

**Affiliations:** 1 Department of Horticultural Sciences, Faculty of Agricultural Science and Engineering, College of Agriculture and Natural Resources, University of Tehran, Karaj P.O. Box 31587-77871, Iran; shirinmoradi@ut.ac.ir (S.M.); asalami@ut.ac.ir (S.A.S.); shokrpour@ut.ac.ir (M.S.); moein.moosavi@ut.ac.ir (M.M.-N.); 2 Photosynthesis Laboratory, Department of Horticulture, Aburaihan Campus, University of Tehran, Tehran P.O. Box 33916-53755, Iran; 3 Copenhagen Plant Science Centre, Department of Plant and Environmental Sciences, University of Copenhagen, Thorvaldsensvej 40, 1871 Frederiksberg, Denmark; cpr@plen.ku.dk; 4 Department of Bioengineering, West Pomeranian University of Technology in Szczecin, 17 Słowackiego Street, 71-434 Szczecin, Poland; jacek.wrobel@zut.edu.pl; 5 Department of Plant Physiology, Institute of Biology, University of Life Sciences SGGW, 02-776 Warsaw, Poland; hazem@kalaji.pl; 6 Institute of Technology and Life Sciences—National Research Institute, Falenty, Al. Hrabska 3, 05-090 Raszyn, Poland

**Keywords:** corm production, chlorophyll fluorescence, light spectra, O-J-I-P transient, photosynthetic functionality

## Abstract

Saffron is a valuable plant and one of the most expensive spices worldwide. Nowadays, there is a tendency to produce this crop in indoor plant production systems. However, the production of saffron is restricted by the need for the reproduction of high-quality corms. In this study, we investigated the effect of different ratios of red (R) and blue (B) light spectra (including 100% B (monochromatic B), 75%, 50%, 40%, 25% B, and 0% B (monochromatic R) on the photosynthetic performance and biomass partitioning as well as morphological and biochemical characteristics of saffron. The growth of flower, root, and corm was improved by increasing the proportion of B to R light. B-grown plants were characterized by the highest photosynthetic functionality with efficient electron transport and lower energy dissipation when compared to R-grown plants. B light directed biomass toward the corms and floral organs, while R light directed it toward the leaves. In saffron, the weight of a daughter corm is of great importance since it determines the yield of the next year. As the ratio of B to R light increased, the daughter corms also became heavier, at the cost of reducing their number, though increasing the proportion of B-enhanced antioxidant capacity as well as the activity of ascorbate peroxidase and catalase while superoxide dismutase activity was enhanced in R-grown plants. In conclusion, B light increased the production of high-quality daughter corms and altered biomass partitioning towards harvestable organs (corms and flowers) in saffron plants.

## 1. Introduction

The dried stigma of saffron (*Crocus sativus* L.) from the Iridaceae family is known as red gold since it is one of the most expensive spices worldwide. Due to its unique flavor and color, saffron is used for culinary, staining, and cosmetics industries. It has medical values because of its chemical components that are used as anti-depression, anti-carcinogenic, and even anti-tumor agents [1,2]. The harvestable part of this crop that is marketed as a valuable spice is stigma, which usually yields around 80 mg per flower [1].

The cultivation of saffron has remained invariant for thousands of years, mostly being produced in farmlands. This form of cultivation leads to several problems such as very low water use efficiency, increasing expenditures on human labor for weed management, irrigation, and flower harvest, lower ability to use mechanization, and most importantly, lower yield per area of cultivation. It seems that shifting from traditional cultivation to closed plant production systems, commonly known as vertical farming, which benefits from mechanization during production processes, would pave the way to improve the profitability of saffron cultivation [3,4,5].

In the last few decades, there is a tendency to produce saffron in protected cultivation systems [2,6,7,8]. Although saffron flowers can be easily produced under greenhouse conditions, the production of high-quality corms under greenhouse conditions is challenging. There are a limited number of studies on vegetative growth and physiological development of saffron corms, highlighting the need for more information to make transferring saffron cultivation from open fields to controlled environments more feasible. Some reports suggested a combined system of handlings between field and controlled environments in a way that the dormancy and flowering phase are carried out in the greenhouse and the vegetative phase in the open field [9,10]. It was reported that saffron plants with high quality and yield could be produced in greenhouse conditions. They introduced a method to increase the flowering period and to extend the saffron harvest period by controlling corms’ storage time and duration [11,12,13].

Corm growth and development is the most important part of the saffron lifecycle since large corms guarantee a high yield in the next year. This stage is highly dependent on environmental conditions such as temperature, light, and water availability [9].

Previous studies presented a clear understanding of the effects of temperature on different stages of the saffron lifecycle. Optimum temperature can be easily maintained in controlled plant production systems that benefit from high-precision sensors. It has also been shown that the lighting environment influences saffron growth; however, there is a large gap in our current knowledge of the proper lighting condition for corm production [9]. In particular, the impact of light quality on biomass allocation toward different organs is largely unknown.

Light is not only a source of energy for photosynthesis but also acts as a signal to modulate plant activities such as photomorphogenesis and other physiological processes such as starch accumulation and secondary metabolite production [14,15,16]. Internal signals that emerge following light exposures regulate growth and development throughout the plant life cycle [14]. Carotenoid biosynthesis, storage, and plastid development are also adjusted by complex light-driven signals [14,17].

Light-emitting diodes (LEDs) have attracted the attention of researchers in recent years due to their benefits over other light sources. The rapid advancement in artificial light technology has increased their application in closed cultivation systems [3]. LEDs with specific wavelengths of light spectra can provide an easy tool for understanding the effects of the light spectrum on plant growth and physiology.

Each waveband of light spectra induces certain responses in plants. Red (R) and Blue (B) lights are more effective for electron excitation in the photosynthetic system since the chlorophyll (Chl) pigments have their highest absorption in those light waveband ranges. Effects of R and B lights, either monochromic or combined, have been widely studied on the growth and physiology of diverse plant species [18,19,20,21]. However, there is still a lack of knowledge on biomass and carbohydrate partitioning toward different organs in bulbous plants such as saffron. Therefore, the present study aimed to investigate photosynthetic performance, biomass and carbohydrate partitioning, and corm production in saffron following exposure to different ratios of B to R light spectra.

## 2. Materials and Methods

### 2.1. Plant Materials and Growth Conditions

Saffron corms (*Crocus sativus* L.) were provided from a traditional saffron production region (Nishabur, Khorasan-Razavi Province, Iran), as one of the major saffron production areas of Iran. After harvesting in late June, equal-weight daughter corms (i.e., 10–12 g) were separated and sorted. They were then dipped into a fungicide solution (0.1% Prochloraz) and dried for 1–2 h. Flower formation was initiated by incubating the corms in the dark at 25 °C at a relative humidity of 85 ± 2% for 90 days, according to [12,13]. Corms were then planted in pots filled with perlite as the culture medium. The rest of the experiment was carried out in a closed LED-equipped controlled-climate room with 10 m^2^ floor area and 2.5 m height, separated by specialized panels to prevent light transmission.

At the beginning of the flowering, corms were fertigated every other day with ½ Hoagland and Arnon [22] nutrient solution using a drip irrigation system. To induce the emergence of flowers, corms were incubated at 17 °C under light conditions provided by blue (B, 400–500 nm), red (R, 600–700 nm), a combination of B and R light, and white (W) LEDs at a photon flux density of 150 μmol m^−2^ s^−1^.

Following flowering, the temperature was set to 15/6 °C (light/dark) with 11/13 h photoperiod for induction of corms, since previous studies showed that the highest sink capacity of corms occurs in this condition [23,24]. 

**Figure 1:**
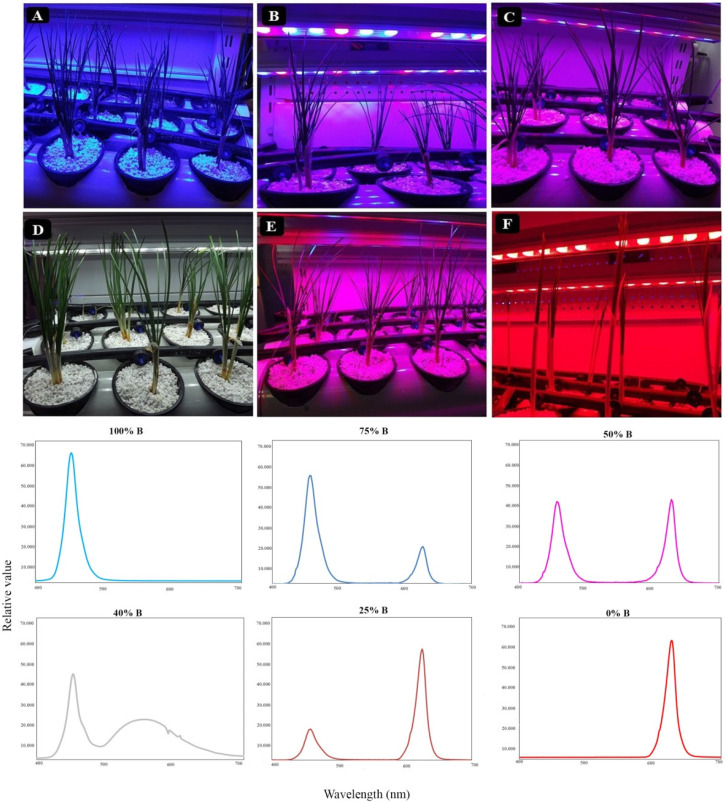
Growth lighting condition of saffron plants as well as spectral distribution of each lighting treatments. Six lighting treatments are shown based on percentage of blue light: 100% B (**A**; monochromatic blue), 75% B (**B**; 75% blue, 25% red), 50% B (**C**; 50% B, 50% R), 40% B (**D**; consisted of 40% in the range of blue and 20% in the range of red), 25% B (**E**; 25% B, 75% R), 0% B (**F**; monochromatic red).

Plants were subsequently subjected to light treatments including monochromatic or combinations of R and B lights in different ratios, including 100% B (monochromatic B), 75% B (75% B, 25% R), 50% B (50% B, 50% R), 40% B (W; consisted of 40% in the range of B waveband and 20% in the range of R waveband), 25% B (25% B, 75% R), 0% B (monochromatic R; Figure 1) provided by LED modules (24 W, OPPLE, Suzhou, China) at a photosynthetic photon flux density (PPFD) of 150 ± 10 μmol m^−2^ s^−1^. The light treatments were separated with a panel wall to segregate light regimes. Light intensity and spectrum were monitored using Fluorpen FP 100-MAX (Photon Systems Instruments, Drasov, Czech Republic) and Sekonic light meter (Sekonic C-7000), respectively. The lighting conditions together with relative spectra of each light treatment are shown in Figure 1.

### 2.2. Morphological and Growth Measurements

Plant height and the number of leaves were recorded weekly. Plant height was measured from the crown of plants to the tip of the longest leaf. After flowering (120 days), some plants were harvested for analysis of plant biomass. Fresh weight (FW) of different parts of plants (including leaf, root, and corm) was recorded immediately after harvesting. Plant tissues were weighed again after oven-drying at 80 °C for 72 h to record their dry weights (DW).

### 2.3. Analysis of Chl Fluorescence and OJIP Test Measurements

Attached, young, fully developed leaves of saffron plants were used for measuring the polyphasic Chl *a* fluorescence (OJIP) transients 60 days after flowering, using a Fluorpen FP 100-MAX (Photon Systems Instruments, Drasov, Czech Republic) following 20 min of dark adaptation. The parameters obtained from the OJIP protocol were calculated according to previous studies [19,20,21,22]. Basic and calculated parameters and their formula are shown in Table 1.

### 2.4. Pigments Measurements

The effect of light conditions on photosynthetic pigment (Chl *a*, *b*, *a* + *b*, and carotenoids) contents was assessed according to the method described by Lichtenthaler (1987). Aliquots of 100 mg of leaf tissue were homogenized by 10 mL of 80% acetone and incubated overnight at 4 °C in darkness. The supernatant was centrifuged at 6000 rpm for 10 min at 25 °C, and absorbance was spectrophotometrically recorded at 646, 663, and 470 nm (PowerWave™ XS Microplate Reader, BioTek Instruments, Inc., Winooski, VT, USA).

### 2.5. Determination of Antioxidant Capacity

Antioxidant activity was detected by the scavenging capacity of the stable 2,2-diphenyl-1 picrylhydrazyl (DPPH) free radical assays according to Sharma’s method [25]. Thus, 12.5 μL of appropriately diluted samples were added to 100 μL of DPPH solution (dissolved in methanol) in a well of a 96-well plate. The absorbance was then measured at 517 nm (PowerWave™ XS Microplate Reader, BioTek Instruments, Inc., Winooski, VT, USA).

### 2.6. Measurement of Antioxidant Enzymes Activity

Activities of ascorbate peroxidase (APX), catalase (CAT), and superoxide dismutase (SOD) as important antioxidant enzymes were assayed 60 days after flowering. APX activity was defined by oxidation of ascorbic acid (AA) at 265 nm (ε = 13.7 mM^−1^ cm^−1^) according to the method described by Nakano and Asada [26]. The enzyme activity was expressed in μmol AA min^−1^ per g of fresh weight.

For the determination of CAT activity, the decomposition of H_2_O_2_ was recorded by the decrease in absorbance at 240 nm. One CAT unit was defined as the amount of enzyme necessary to decompose 1 mM min^−1^ H_2_O_2_. Therefore, the CAT activity was expressed as U g^−1^ FW min^−1^ [27].

The activity of SOD was determined according to the method described by Dhindsa et al. [28]. The method is based on the ability of SOD to inhibit the photochemical reduction of nitro blue tetrazolium (NBT). Briefly, the reaction was started by switching on the light Fluorescent lamp (50 W, 60 cm) and was allowed to run for 10 min. The reaction was stopped by turning off the light, and the tubes were covered using a black cloth. The absorbance of the reaction mixture was recorded at 560 nm.

### 2.7. Determination of Carbohydrates in Leaves and Corms

For assaying the carbohydrate concentration, both young leaves and daughter corms were collected 120 days after flowering. The total soluble carbohydrates were analyzed using the anthrone procedure in which samples were ground in liquid nitrogen, and 0.2 g tissue was sampled and blended with 7 mL of 70% ethanol (*w*/*v*) for 5 min on ice and centrifuged (6700× *g*) for 10 min at 4 °C. After adding 200 mL of the supernatant to 1 mL of an anthrone solution (0.5 g anthrone, 250 mL 95% H_2_SO_4_, and 12.5 mL distilled water), the absorbance was spectrophotometrically recorded (PowerWave™ XS Microplate Reader, BioTek Instruments, Inc., Winooski, VT, USA) at 625 nm according to van Doorn [29]. The determination of starch amount was also completed using the sugar–anthrone–sulfuric acid reaction based on the method of McCready [30].

### 2.8. Statistical Analysis

The data were statistically evaluated by the ‘completely randomized design’ method using six different light treatments with twenty plants as replicates in each light treatment. Individual plants were taken as independent replicates. Six (for all measurements except morphological traits) or nine (morphological traits) replicates in each treatment were considered for subsequent analysis. The data were subjected to analysis of variance (ANOVA), and means were compared using Duncan’s multiple range test at a significance level of 0.01 using the SAS software (Statistical Analysis System, version 9.4).

## 3. Results

### 3.1. Plant Morphology and Architecture

Although B and R lights are the main light spectra for the absorption spectra of chlorophyll, photosynthesis, and growth, studies on their roles on the growth, development, and biomass partitioning of bulbous plants are scarce. In the present study, to investigate the influence of B light on the growth of different organs of saffron, 90 days after incubation at 25 °C under dark conditions, corms were incubated at 17 °C under the light treatments for flower emergence. Flowering occurred 20–28 days after light treatment. The number of flowers per corm, flower, and stigma FW and DW were measured. Significant differences among treatments were found on the flower number per corm, flower, and stigma FW and DW (Table 2). The highest number of flowers per corm, flower FW, and DW were observed in B-light-grown plants. The highest stigma FW and DW were also observed under B light.

According to the results (Table 2), the flower number of saffron plants grown under B light was almost four times higher than their values in R-light-grown plants. However, no significant differences were detected among treatments containing less than 50% B (i.e., 40% B, 25% B, and 0% B). The highest flower FW and DW were noted under monochromatic B with significant differences with other treatments. Yet, regarding stigma FW and DW, no significant change was detected under monochromatic B and 50% B. An intermediate DW and FW were also noted under 75% B. While stigma FW under monochromatic B was two-fold, their stigma DW was four-fold, compare to those of monochromatic R, revealing higher water content of R-grown stigma. 

To investigate the effect of light spectra on saffron vegetative growth, plants were sampled for biomass partitioning analysis 120 days after flowering (in this time, daughter corms were fully developed). Results showed that all characteristics were significantly influenced by the different ratios of B to R light (Table 3). R light positively affected leaf growth, revealed by the highest number of leaf and highest leaf FW and DW, which were observed in the high percentage of R proportion, especially under monochromic R light (0% B). However, R light negatively affected root growth. Root characteristics were influenced synergically by a higher ratio of B to R light. The highest length of root (3.47 cm) and highest root FW (46.4 mg) and DW (0.44 mg) were observed under 100% B-light treatment. 

These results indicate that R light has a positive effect on above-ground elongation, while B-light application results in the production of compact plants with shorter leaves. Morphological characteristics related to leaves such as the number of leaves, leaf FW and DW, and leaf length increased under monochromatic R, while B promoted morphological characteristics of roots such as root length, root FW, and DW. The highest number of leaves was noted under 25% B, though with no significant difference with those of monochromatic R (0% B). A minimum number of leaves was also detected in monochromatic B- and 75% B-light-grown saffron plants. The trend for leaf FW, DW, and length was also similar. Saffron roots, on the other hand, thrived under treatments with a high proportion of B light. Root FW and DW of plants grown under monochromatic B light in comparison to those of monochromatic R was 2.7 and 3.1 times higher, respectively. Yet, the longest roots were observed under 50% B. 

**Figure 2:**
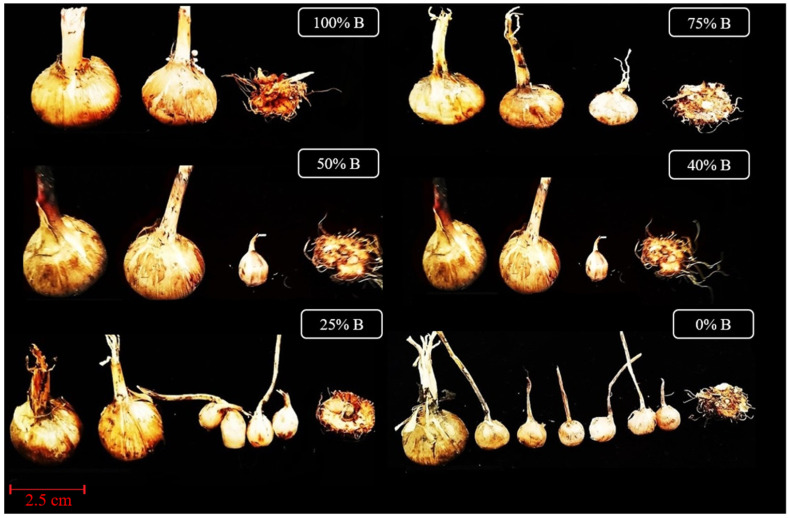
Saffron corms produced under different ratios of Blue (B) light in the overall spectral composition, including 100% B, 75% B, 50% B, 40% B, 25% B, 0% B (see the spectrum in Figure 1), with 150 ± 10 μmol m^−2^ s^−1^ PPFD. In each treatment, the new corms are arranged based on their weight from largest to smallest together with the last one, which is the remains remainingof the mother corm.

Finally, corm production was analyzed as the main goal of this study because it is the most important part of saffron vegetative growth. Corm production and daughter corm’s characteristics were significantly (*p* ≤ 0.01) affected by light spectra (Table 4). As the ratio of B to R light increased, the daughter corms also became heavier, though at the cost of reducing their number. B light significantly increased the biomass of the new corms, while because of the reduction of new lateral root buds, the number of daughter corms was reduced. Overall, decreasing the ratio of B to R led to the production of small new corms (Figure 2). The heaviest daughter corms were observed under monochromic B light (100% B), while the maximum number of corms was observed in monochromic R-grown plants (0% B). 

In saffron, the weight of the daughter corms is of great importance since it determines the yield of the next year [9]. Accordingly, 50% B treatment had the highest efficiency among the treatments by producing the appropriate number of heavy daughter corms.

### 3.2. Carbohydrate Contents and Biomass Partitioning

**Figure 3:**
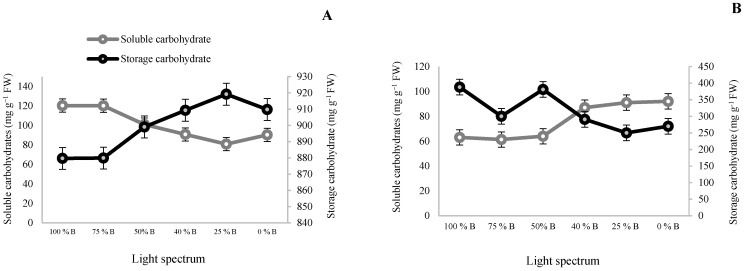
Concentration of soluble and storage carbohydrates in the leaves (**A**) and corms (**B**) of saffron plants grown under different ratios of blue (B) to Red (R) light in the overall spectral composition, including 100% B, 75% B, 50% B, 40% B, 25% B, 0% B (see the spectrum in Figure 1), with 150 ± 10 μmol m^−2^ s^−1^ PPFD. Bars represent mean value ± standard deviation (*p* ≤ 0.01).

To investigate the effect of different light spectra on the translocation and accumulation of carbohydrates in different organs, the amount of soluble and storage (starch) carbohydrates in both leaf and corm tissues were measured. The results showed that carbohydrate content was significantly influenced by different ratios of B to R lights (Figure 3). The results of the carbohydrate content in leaves showed a linear relationship between increasing starch content and decreasing B-light percentage. Except for monochromatic R light (0% B), the reduction in leaf starch was detected by increasing the percentage of B light. The highest amount of starch was observed in 25% B light (91.9 mg g^−1^ FW) treatment, and the lowest amount was observed in 0% B or 75% B (87.9 mg g^−1^ FW). On the contrary, soluble carbohydrate content showed an increasing trend with the increase in the B-light percentage. The highest soluble carbohydrate was detected in 100% B-light-grown leaves by 120.33 mg g^−1^ FW, which was 1.5 times higher than R light (0% B). The results obtained from the carbohydrate contents in corms differed from those obtained from the leaves. The corms of plants grown under treatments with less than 50% B light were not significantly different but showed significant differences with the corms of plants grown under treatments above 50% B light (50, 75, 100% B). Their soluble carbohydrate content was also more than in the corms of plants exposed to 50% B light. The lowest amount of soluble carbohydrates was recorded in corms grown under 100% B light (63.05 mg g^−1^ FW). Soluble carbohydrates produced in corms of plants exposed to monochromatic R light (0% B) were 1.5 times more than those of 100% B. Except for 75% B, an increasing trend was detected between starch content and the percentage of B light. The highest amount of starch was recorded in the corms grown under 100% B-light treatment (378.96 mg g^−1^ FW); the starch content was 1.5 times higher than the R-light treatment.

**Figure 4:**
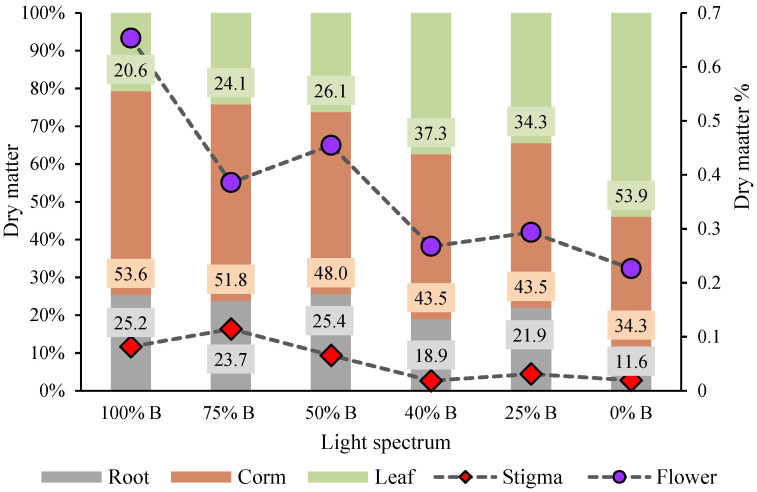
Biomass partitioning of saffron plants grown under different ratios of Blue (B) to Red (R) light in the overall spectral composition, including 100% B, 75% B, 50% B, 40% B, 25% B, 0% B (see the spectrum in Figure 1), with 150 ± 10 μmol m^−2^ s^−1^ PPFD. Due to the allocation of less than 1% of total dry matter to the reproductive organs, flower and stigma dry matters are shown by purple circles and red rhombus, respectively, showing their values by the right *Y*-axis.

Understanding the effects of different ratios of B to R on biomass partitioning in saffron plants, as well as the relationship between carbohydrate transfer and also distribution of photo-assimilates in different organs in the developmental stage of new corms, is of great importance. Therefore, the DW of all parts of the plants was measured and divided by the total dry matter (Figure 4). Figure 4 clearly shows that plants have allocated more biomass to the aerial parts by reducing the percentage of B to R light. Moreover, plants grown under monochromatic R light produced 2.6 times more biomass in aerial parts in comparison with the B-grown plants. The percentage of biomass allocation to the underground organs (roots and corms) in the plants grown under monochromatic B was almost two-fold when compared to R-grown plants. In plants grown under treatments with a high ratio of B to R (i.e., 50, 75, and 100% B), more than half of the biomass was partitioned in the underground parts, especially in the new corms. The highest percentage of partitioning to underground organs (78.8% to roots and corms) was recorded in the plants grown under monochromatic B light, out of which 53.6% resulted from corm production and 25.2% from new roots. On the other hand, a higher proportion of R light in the spectrum caused partitioning of biomass towards the aerial part, especially to the leaves (~54%). The highest percentage of biomass partitioning towards flower and stigma was recorded in plants grown under 100% Blight which was almost three and four times more than their biomass in plants grown under 0% B light, respectively. 

### 3.3. Photosynthetic Parameters

The energy content of different light spectra is absorbed by the Chl pigments, which directly influence the electron transport chain of the photosynthetic apparatus. Therefore, to investigate the effects of different ratios of B to R light in the overall spectrum on the photosynthetic functionality, polyphasic Chl *a* fluorescence (OJIP) on the dark-adapted leaves of saffron plants grown under different proportions of B light was analyzed.

**Figure 5:**
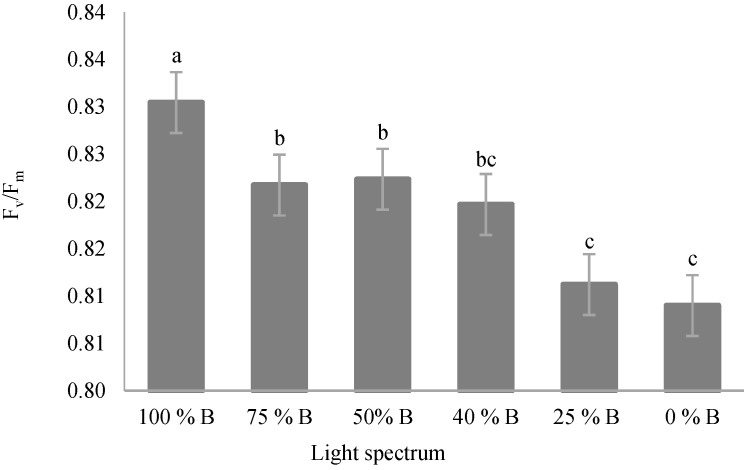
The maximum quantum yield of PSII (F_v_/F_m_; equation in Table 1) of saffron plants grown under different ratios of Blue (B) to Red (R) light in the overall spectral composition, including 100% B, 75% B, 50% B, 40% B, 25% B, 0% B (see the spectra in Figure 1), with 150 ± 10 μmol m^−2^ s^−1^ PPFD. Different letters indicate that values are significantly different at *p* < 0.01 according to Duncan’s multiple range tests. Bars represent mean value ± standard deviation.

The maximum quantum yield of PSII (F_v_/F_m_) was significantly affected by different light treatments. Results showed that the F_v_/F_m_ significantly decreased by the decrease in the ratio of B to R light in all treatments (Figure 5). The highest F_v_/F_m_ (0.83) was recorded under 100% B-light treatment. An intermediate F_v_/F_m_ was also recognized under 75% B, 50% B, and 40% B, with no significant differences among them. The lowest F_v_/F_m_, on the other hand, was detected in 25% B- and 0% B-light-grown plants.

**Figure 6:**
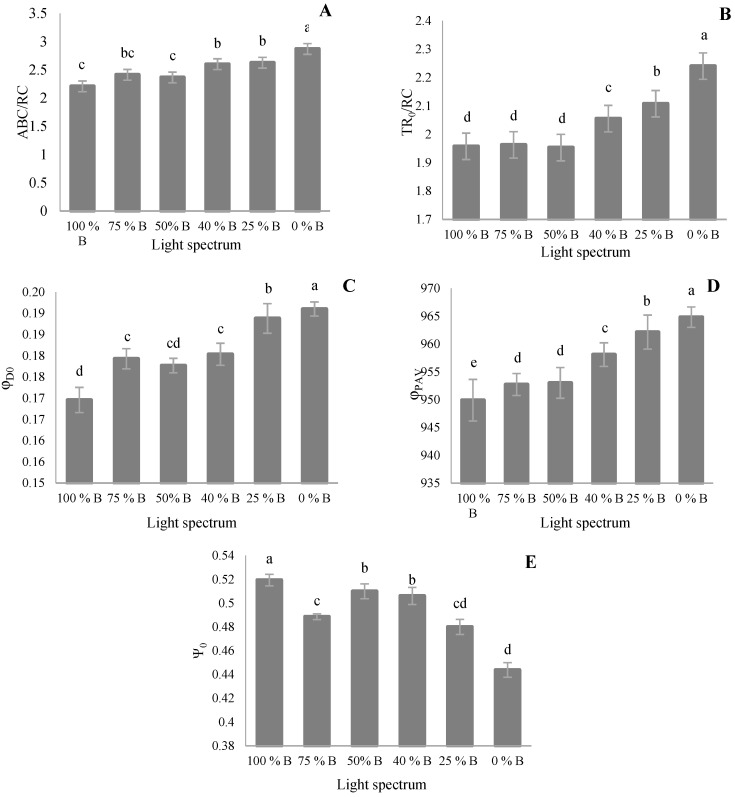
Specific energy fluxes per reaction center (RC) for energy absorption (**A**; (ABS/RC)), trapped energy flux (**B**; (TR_0_/RC)), electron transport flux (**C**; (ET_0_/RC)), and dissipated energy flux (**D**; (DI_0_/RC); equations in Table 1) of saffron plants grown under different ratios of Blue (**E**) light in the overall spectral composition, including 100% B, 75% B, 50% B, 40% B, 25% B, 0% B (see the spectrum in Figure 1), with 150 ± 10 μmol m^−2^ s^−1^ PPFD. Different letters indicate that values are significantly different at *p* < 0.01 according to Duncan’s multiple range tests. Bars represent mean value ± standard deviation.

Specific energy fluxes per reaction center (RC) were measured based on the output of fast inductions of Chl-*a* fluorescence. Parameters such as ABS/RC, TR_0_/RC, and DI_0_/RC (Figure 6A,B,D) decreased by increasing the B-light proportion. The highest values of these parameters were observed in plants grown under 0% B. In the case of TR_0_/RC, significant differences were not detected among plants exposed to lights containing more than 50% B light. TR_0_/RC increased by increasing the percentage of R light. The highest TR_0_/RC was recorded in plants exposed to monochromatic R light (Figure 6B). The ratio of ET_0_/RC also reduced with decreasing B proportion in overall light spectra (Figure 6C). These results indicate the notable importance of B light on maintaining the normal electron transport in the PSII apparatus.

**Figure 7:**
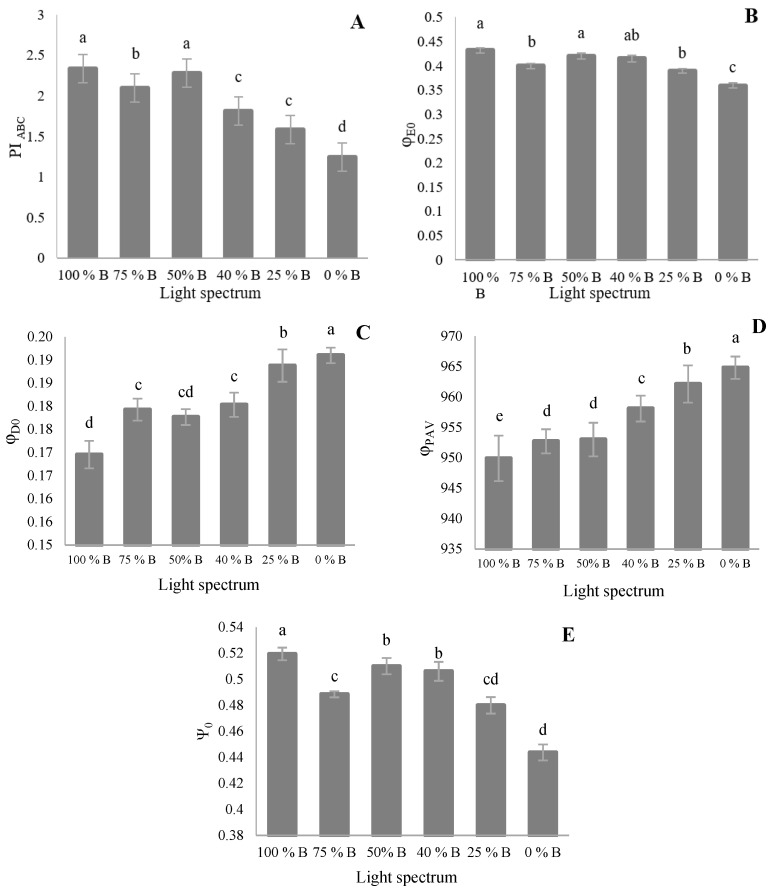
Performance index on the absorption basis (**A**; (PI_ABS_)), the maximum quantum yield of primary photochemistry, (**B**; (φ_E0_)), quantum yield of energy dissipation (**C**; (φ_D0_)), average (from time 0 to t FM) quantum yield for primary photochemistry (**D**; (φ_PAV_)), and the probability that a trapped exciton moves an electron in the electron transport chain beyond Q_A_^–^(**E**; (Ψ_0_); equation in Table 1) from the fluorescence transient exhibited by leaves of saffron plants grown under different ratios of Blue (B) to Red (R) light in the overall spectral composition, including 100% B, 75% B, 50% B, 40% B, 25% B, 0% B (see the spectrum in Figure 1), with 150 ± 10 μmol m^−2^ s^−1^ PPFD. Different letters indicate that values are significantly different at *p* < 0.01 according to Duncan’s multiple range tests. Bars represent mean value ± standard deviation.

The performance index on the absorption basis (PI_ABS_) represents the validity index of PSII. This parameter increased significantly with high B-light proportions, representing the highest value in 100% B treatment, though with no significant differences with 50% B (Figure 7A). 75% B-grown plants also ranked second, higher than those of 40% B- and 20% B-grown plants. The quantum yield of energy dissipation (φ_D0_; Figure 7C) and average quantum yield for primary photochemistry (φ_PAV_; Figure 7D) gradually decreased by increasing B-light proportions. Quantum yield of energy dissipation (φ_D0_) was the highest in plants grown under monochromatic R (0% B), followed by those of 25% B, but no significant difference was noted in φ_D0_ value among seedlings grown under 40%, 50%, and 75% B. The least φ_D0_ value was also observed under 100% B. This trend almost similarly occurred for φ_PAV_. The least value of PI_ABS_ was, on the other side, recorded in 0% B. The probability that a trapped exciton moves an electron in the electron transport chain beyond Q_A_ (Ψ_0_; Figure 7E) was gradually increased by increasing the ratio of B to R light, though 75% B provided an exception, not having significant differences with 25% B.

### 3.4. Pigment Content

**Figure 8:**
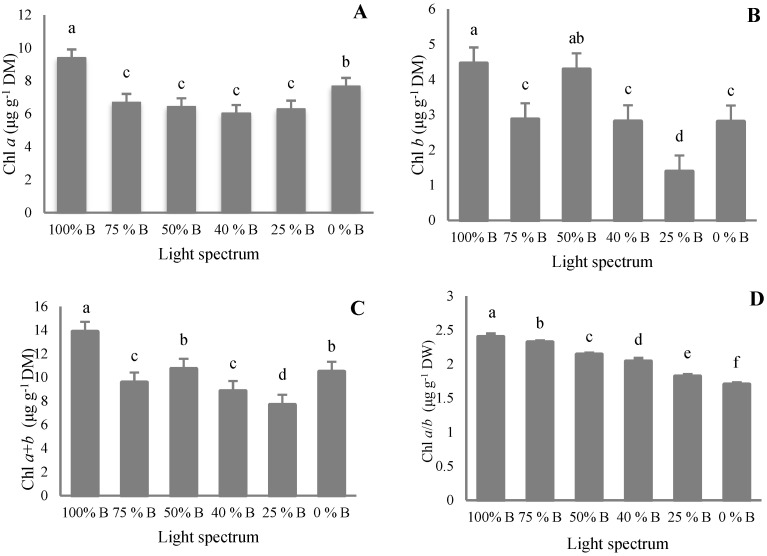

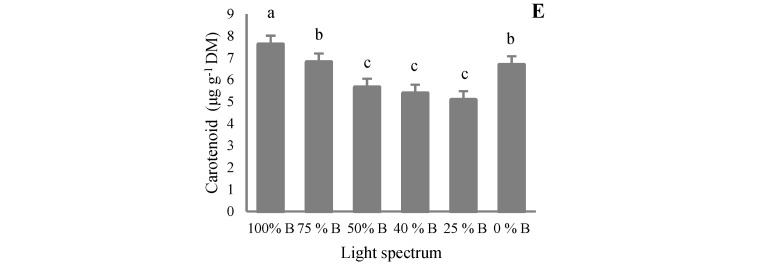
Chlorophyll *a* (**A**; Chl *a*), Chlorophyll *b* (**B**; Chl *b*), total chlorophyll (**C**; Chl *a* + *b*), chlorophyll *a*/*b* ratio (**D**; Chl *a*/*b*) and carotenoid (**E**) accumulation of saffron plants grown under different ratios of Blue (B) to Red (R) light in the overall spectral composition including 100% B, 75% B, 50% B, 40% B, 25% B, 0% B (see the spectrum in Figure 1), with 150 ± 10 μmol m^−2^ s^−1^ PPFD. Different letters indicate that values are significantly different at *p* < 0.01 according to Duncan’s multiple range tests. Bars represent mean value ± standard deviation.

Pigment concentration was analyzed to investigate the effects of different ratios of B to R light in the overall spectrum on the pigment accumulation in the leaves of saffron plants. According to the analysis of the obtained data, the highest content of Chl *a*, Chl *b*, Chl *a* + *b,* and Chl *a*/*b* was observed in 100% B treatment (Figure 8A–D). The results showed that the amount of Chl *a*, Chl *b*, Chl *a*/*b*, Chl *a* + *b*, and carotenoids in monochromatic light treatments were higher than in dichromatic treatments. The highest amount of Chl *a* was recorded in monochromatic B light followed by monochromatic R. The combination of R and B light treatments showed the least concentration of this pigment with no significant difference among these four treatments (i.e., 75%, 50%, 40%, and 25% B light). The results obtained from Chl *b* data did not show a clear trend between the percentage of B light and the concentration of Chl *b* (Figure 8B). The highest concentration of Chl *b* was observed in plants grown under 100 and 50% B light. The concentration of Chl *b* in the leaves grown under 100% B light was 3.2 times higher than leaves grown under 25% B light. The results indicated that there is a positive interaction between increasing B to R light ratio and Chl *a*/*b* ratio (Figure 8D). Chl *a*/*b* ratio ranked first in plants exposed to 100% B light, being 1.5 times more than that of 0% B light treatment. 

Carotenoid accumulation increased in plants grown under monochromatic B light, followed by 75% B and monochromatic R. On the other hand, leaves exposed to 50, 40, and 25% B showed the least amount of carotenoid without any significant differences (Figure 8E).

### 3.5. Antioxidant Capacity

**Figure 9:**
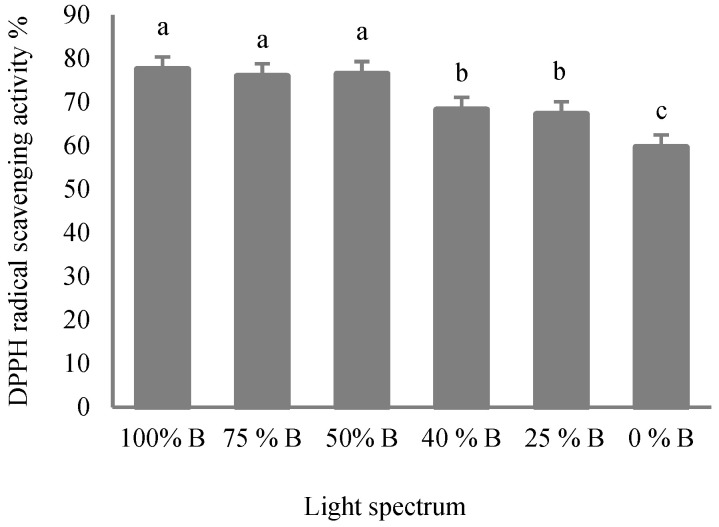
Antioxidant capacity of saffron plants grown under different ratios of Blue (B) to Red (R) light in the overall spectral composition, including 100% B, 75% B, 50% B, 40% B, 25% B, 0% B (see the spectrum in Figure 1), with 150 ± 10 μmol m^−2^ s^−1^ PPFD. Different letters indicate that values are significantly different at *p* < 0.01 according to Duncan’s multiple range tests. Bars represent mean value ± standard deviation.

Light spectra significantly affect the antioxidant capacity of plants [14]. The estimation of antioxidant capacity was completed by determining DPPH scavenging capacity. In this mechanism, antioxidant inhibits lipid oxidation [31]. In the present study, plants exposed to high ratios of B to R light (i.e., treatments containing more than 50% of B) showed positive effects on the stable DPPH free radical scavenging activity (Figure 9). Thus, plants grown under these treatments showed higher antioxidant capacities than those grown under lesser proportions of B (i.e., 40 and 50% B). The least percentage of DPPH activity was also noted in monochromatic R- grown plants.

### 3.6. Antioxidant Enzymes Activity

**Figure 10:**
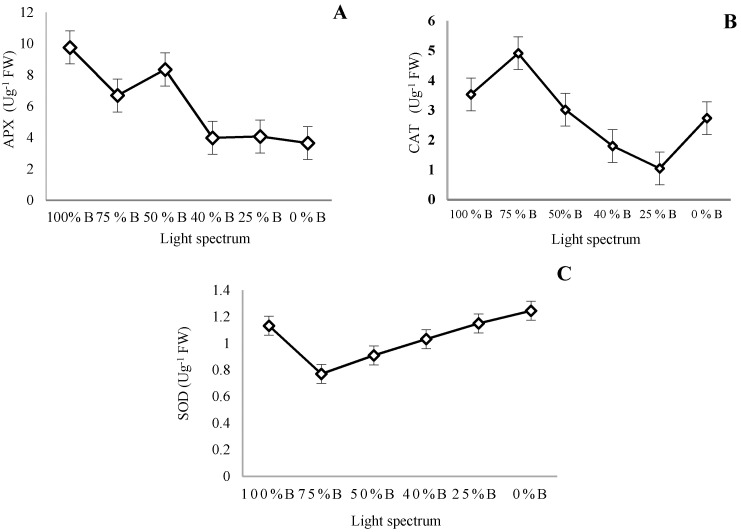
Ascorbate peroxidase (**A**; APX), catalase (**B**; CAT), and superoxide dismutase (**C**; SOD) activity of saffron plants grown under different ratios of Blue (B) to Red (R) light in the overall spectral composition, including 100% B, 75% B, 50% B, 40% B, 25% B, 0% B (see the spectrum in Figure 1), with 150 ± 10 μmol m^−2^ s^−1^ PPFD. Bars represent mean value ± standard deviation (*p* ≤ 0.01).

Different ratios of B to R light significantly influenced the activity of APX, CAT, and SOD enzymes in contrasting ways (Figure 10A–C). The activity of APX showed a declining trend with the reduction of the B to R light ratio. The highest APX activity, which was 2.7 times more than APX activity in the plant grown under 0% B light, was detected in plants exposed to 100% B (Figure 10A). Regardless of two monochromic treatments (i.e., monochromatic R and B), a gradual decrease in CAT activity was recorded with the reduction of the B to R light ratio (Figure 10B). The highest CAT activity was recorded in plants grown under 75% B light, which was 4.7 times more than that of 0% B light. In contrast, SOD activity decreased with an increasing ratio of R to B light. The highest SOD activity was recorded in plants exposed to monochromatic R light. SOD activity in the plant grown under R light was 1.5-fold compare to 75% B light (Figure 10C).

## 4. Discussion

### 4.1. Growth and Morphology of Saffron Plants Were Influenced by Light Quality

Photomorphogenesis is a plant strategy facilitating plant adaptation to the environment. This process is directly influenced by light, which is regulated by light receptors such as phytochromes (PHY) that are sensitive to R light, cryptochromes (CRY), and phototropins (PHOT), which are sensitive to B light. The signals produced by the light receptors induce physiological and biochemical changes in different growth pathways [32]. 

As expected, in this study, a clear morphological response was observed in different parts of the plants grown under different lighting conditions. High ratios of B to R light caused the generation of more flowers with the highest flower FW and DW. A positive effect of B light was recorded on stigma FW and DW. There are shreds of evidence that B light promotes flowering by the interaction between CRY and flowering genes such as FLOWERING LOCUS T (FT) and CONSTANS (CO) [32]. Previous studies also showed a positive effect of B light on the flowering of bulbous plants such as tulip [18] and other plant species such as pepper [31]. Additionally, the same effect of R light on decreasing flowering has been reported in Indian mustard and basil plants [33]. 

In the present study, a high number of leaves and increased length of leaves were observed in plants grown under R light. On the contrary, B light caused plant compactness. It has been known that CRY1 (sensitive to B) represses hypocotyl cell elongation and induces cell enlargement and development by modulating the biosynthesis and transport of endogenous auxins such as Indole-3-Acetic Acid (IAA) and their analogs [34]. Similar findings have been reported in other studies as well. For instance, Ouzounis et al. [35] reported that by increasing B light dosage, hypocotyl length and plant height reduce. The R light, as a primary light source, affects stem elongation via up-regulating Gibberellins (GAs) levels and by blocking negative-GA signaling components in the tissue, resulting in elongation of stem internodes, while B light up-regulates the negative-GA signaling components, leading to compactness without morphological abnormalities [36]. In cucumber, increasing B light by up to 75% caused a significant reduction in hypocotyl elongation and plant length [37]. 

The results of the current study clearly show the positive effects of B light on the length, FW, and DW of the roots. It was also reported that root elongation and development are regulated by CRY and PHOT [34]. Therefore, cytoplasmic CRY1 promotes root elongation by modulating the auxin efflux rate, while PHOT represses the lateral bud growth and its number. It should also be noted that PHOT2 only acts when a high ratio of B to R light is applied [15]. 

In a study on potatoes, B light increased the number of adventitious roots and the root DW compared to shoot DW [38]. Similarly, the longest root and highest root DW were observed after treatments with a high percentage of B light.

The weight of the mother corms is known as a determining factor in saffron production. It has been shown that the size of the mother corm not only has a significant effect on extending harvest time, flower number, stigma FW, and DW, but it also has a significant effect on vegetative development and the production of daughter corms in the subsequent year [8,9,39,40]. Accordingly, the number, FW, DW, and diameter of daughter corms per mother corm were measured in this study. The results showed that B light improved the weight and decreased the number of the daughter corms at the same time. On the other hand, a low ratio of B to R light led to an increase in the number and reduced the weight of daughter corms. It seems that B light induces the apical dominance on corm buds and inhibits the outgrowth of lateral buds.

Apical dominance is regulated by complex environmental and physiological signals such as light quality, auxin, cytokinin (CK), strigolactone (SL) level, and sugar availability status. It was reported that a high level of B light increased the accumulation of auxin in the shoot apex and induced apical dominance in pea plants [36]. The results of the current study are in accordance with this hypothesis. 

Shoot and root architecture is a plant adaptation strategy that is particularly regulated by light quality [34]. It has been reported that R light promotes the bud outgrowth and shoots branching by increasing the biosynthesis of GAs [34,36]. In this study, the results of biomass partitioning showed that under the high ratio of B to R light, most of the biomass was partitioned to the underground parts of the plants, especially in the new corms, while having a higher proportion of R light in the spectrum caused partitioning of biomass towards the aerial part, especially to the leaves. Production of new corms with a high weight and large size is a milestone in the successful production cycle of saffron. In the current study, B light improved the weight of the daughter corms, while it decreased their numbers. According to the obtained results, 100% B light made the daughter corm FW and DW 2.62 and 6.7 times more than the daughter corm FW and DW under 0% B light, respectively (Table 4). In contrast, a high proportion of R light promoted the production of small daughter corms. The number of daughter corms in 0% B-light treatment was two-fold compared to 100% B light. Reports on the effects of light spectra on the development of underground organs in different species are scarce. In a few of the cases, Lian et al. [41] suggested that it is possible to affect in vitro growth of lily bulblets during the enlargement stage by manipulating the quality of light. They found that B and R lights (50% B: 50% R) induced better growth of bulblets, with bigger size and higher FW and DW compared with bulblets under other lighting conditions. Additionally, restricted growth and lower dry matter accumulation in plants grown under R LEDs alone may be related to a lower CO_2_ assimilation rate, as reported before in wheat [42].

### 4.2. B Light Directed Carbohydrates and Biomass towards the Underground Parts of Saffron

To understand the effect of different light spectra on the translocation and accumulation of carbohydrates in different organs, the amount of soluble and insoluble (starch) carbohydrates in both leaf and corm tissues were measured. The highest concentrations of starch (both in corms and leaves) were detected in the treatments containing a high ratio of B to R light, while the amount of soluble carbohydrate in these organs increased by the increase in the proportion of R light. These results indicated that B light induced the storage of carbohydrates through increasing starch accumulation in the developmental stage of daughter corms. On the other hand, R light led the plants to focus on leaf growth and starch accumulation in the leaves.

In agreement with this study, it has been reported that R light inhibits the transport of carbohydrates from leaves to sink organs that cause a decrease in photosynthesis because of the high amount of carbohydrates in the leaves [15]. Additionally, Samuoliene et al. [43] found an increase in the number of carbohydrates in the leaves of strawberry plants grown under 50% R and 50% B lights. Furthermore, transplants of cucumber, tomato, and sweet pepper showed an increase in FW and DW and biomass accumulation under B light. They indicated that all spectra combined with additional B light increased the FW of transplants [43]. Lettuce, spinach, kale, basil, and sweet pepper plants cultured under 100% R light showed lower FW and DW compared to those grown under a combination of R and B lights [31]. Saebo et al. [44] suggested that lower biomass accumulation under R light is due to the enhancement of starch content by inhibiting the translocation of photosynthates out of the leaves, causing negative effects on the growth of other parts of the plants such as flowers.

### 4.3. Photosynthetic Functionality of Saffron Plants Down-Regulated by R Light

In this study, to understand the effect of light spectra on the photosynthetic performance of saffron plants grown under different ratios of B to R, the parameters obtained from the OJIP test were analyzed. It has been shown that healthy plants have an F_v_/F_m_ value of around 0.83 [45,46,47]. In this study, plants grown under a high percentage of B light showed the optimal value of F_v_/F_m_, while the lowest value was observed in 0% B (monochromic R light). These results indicated the positive role of B light on the efficiency of PSII of the saffron plants. 

Consistent with our results, it has been indicated that B light improves the performance of photosynthesis through limiting energy dissipation and elevating electron transport in the electron transport chain of the photosynthetic apparatus [19]. Chen et al. [48] suggested that a decrease in F_v_/F_m_ by R light is due to the reduction of photochemical activity caused by the inactivation of the PSII reaction centers and damage to the D_1_ protein. The better photosynthetic performance in plants grown under B light and the negative effects of R light on the performance of electron transport flow of photosynthetic apparatus have also been reported in cucumber [49,50], marigold [51], basil [52], and chrysanthemum [21].

DI_0_/RC and φ_D0_ show the energy dissipation in PSII, so the high value of these parameters reveals the high conversion of energy, mainly to heat. Plants perform this to protect cells against light-induced damage [53]. These parameters increased in plants grown under a high proportion of R light, indicating down-regulation of the photosynthesis efficiency by reduction of the B to R light ratio. In accordance, an increase in φ_D0_ and DI_0_/RC in plants grown under R light was reported in basil [52], marigold [51], and chrysanthemum [21]. ABS/RC increased in plants grown under a high proportion of R light. The increase in ABS/RC could be attributed to either the inactivation of PSII centers or an increase in the size of the receptors [54,55,56]. In accordance with our result, high values of ABS/RC in plants grown under R light treatment have been reported in other plant species [51,52]. Therefore, for keeping normal photosynthetic functionality, a certain ratio of B to R light in the overall spectra is needed [49]. In this study, the highest ET_0_/RC was observed in plants grown under 100% B, mainly because of higher inhibition of re-oxidation of Q_A_^–^ to Q_A_ [55,57]. PI_ABS_ amalgamates the energy fluxes from the early step of the absorption process until the plastoquinone reduction [55]. In many studies, it has been suggested that under different environmental conditions, PI_ABS_ is the most sensitive parameter for measuring photosynthetic performance in plants under abiotic stresses [45,57,58,59]. PI_ABS_ represents the function of PSII; thereby, decreases in PI_ABS_ could be due to suppression of electron transfer as a result of the decrease in PSII functionality [60]. In this study, a high value of PI_ABS_ was recorded in plants grown under high percentages of B light, indicating the best performance of PSII in the B-light-grown saffron plants.

### 4.4. Pigment Accumulation in Saffron Occurred by B Light Exposure

As the main photosynthetic pigments in plants, chlorophylls (Chls) and carotenoids function in the plant physiological processes, including light-harvesting, photo-oxidation, plant coloring, and provide nutritional benefits as a precursor to essential vitamins and antioxidants [14]. Photosynthetic pigment formation is particularly regulated by light spectra [17]. All of these demonstrate the importance of light quality on the optimization of phytochemical concentration in plant species [31]. In this study, plants grown under 100% B produced the highest Chl *a*, *b*, and Chl *a* + *b* contents. Treatments with a high proportion of B light also raised the Chl *a*/*b* ratio. It has been known that B light increased the biosynthesis of 5-aminolevulinic acid (ALA), which is a primary substrate of Chl [61]. B light also up-regulated the expression of important genes in the Chl biosynthesis pathway, such as *MgCH*, *GluTR,* and *FeCH*. B light simulates the chloroplast development and increases photosynthetic performance by increasing the Chl content and Chl *a*/*b* ratio [14,32]. The positive effect of B light on enhancing Chl concentration has been reported in diverse plant species, including cucumber [49], cabbage seedlings [62], non-heading Chinese cabbage [63], lettuce, cucumber, tomato, radish, pepper [64], and basil [52]. However, there are some reports indicating different impacts of light on the accumulation of pigments [35]. In contrast, it has been reported that Chl *a*, Chl *b*, and Chl *a* + *b* concentrations increase in lettuce, basil, spinach, kale, and pepper in high proportions of R light [31].

Carotenoids strongly absorb light in the B region, with maximum peaks occurring at 454 and 448 nm [17]. It has been reported that B light can act synergistically on biosynthesis and accumulation of carotenoids. For instance, the accumulation of carotenoids was provoked by B light during the vase life of carnation plants [65]. It has also been shown that plants contain high carotenoid concentrations when they are grown under high B to R ratios [31], which confirms the findings of the current study on the positive effect of B light on increasing carotenoid concentration.

### 4.5. Antioxidant Enzymes and Oxidative Damage Are Influenced by Light Quality in Saffron

Carotenoids are parts of the antioxidative defense system in higher plants [66]. Elevation of antioxidant capacity by B light has also been reported in different plant species [19,31]. It has been suggested that a high amount of antioxidant capacity may be due to the high concentration of the pigments, especially carotenoids, in plants grown under the higher B light ratios [31,66]. 

Superoxide dismutase (SOD) acts as the first line of defense that converts free radical oxygen into H_2_O_2_; then, the generated H_2_O_2_ is converted into H_2_O by CAT and APX. Accordingly, CAT and APX have the same activity [67]. B light is known as a signal that modulates plant responses to biotic stresses [68]. In the present study, higher activity of APX and CAT was detected in plants grown under treatments with a high B to R ratio, while this lighting environment decreases the activity of SOD. Other studies also indicated that B light positively affects ascorbic acid synthesis [19,69,70,71]. These reports explain the positive effect of B light on increasing the antioxidant capacity of plants. It has been suggested that B light induces the activity of CAT and APX in some plant species, such as carnation [19], lettuce [71,72], *Rehmannia glutinosa*, and *Stevia rebaudiana* [73]. Bayat et al. [57] showed a positive correlation between SOD activity and H_2_O_2_ production, while CAT and APX negatively regulate H_2_O_2_ production. The accumulation of H_2_O_2_ following stress could be attributed to an increase in SOD activity and a decrease in CAT and APX activities. They also indicated a negative relationship between storage carbohydrate content and SOD activity [57]. This finding can explain the high activity of SOD under R light treatment with the lowest amount of storage carbohydrates.

## 5. Conclusions

As the main source of nutrient supply, corms play a vital role in the saffron life cycle. The size of the corms and their weight not only determine the yield of stigmas and the quality of daughter corms but also affect the duration of the harvest period. This study, for the first time, investigates the effect of different ratios of two important light spectra for plant photosynthesis, R and B lights, on the biomass partitioning, photosynthetic performance, and biochemical characteristics of saffron. A high ratio of B light increased flower production with a higher stigma yield and also led to the emergence of compact plants. Furthermore, this region of light spectra improved the photosynthetic performance of saffron plants by facilitating electron flow between PSII and PSI. The high proportion of B to R positively affected corm production with the highest corm yield and carbohydrate accumulation of saffron plants. B light stimulated and increased starch accumulation in the corm organ. This can be associated with the ability of B light to keep high photosynthetic performance. An increase in the ratio of B to R light induces the plants to produce underground organs, leading to the production of heavy daughter corms, while high R light ratios stimulated the production of lateral buds and led the plant to produce leaves. B light caused an increase in antioxidant capacity and induced the activity of CAT and APX. Based on the findings of the present study, B light leads to better photosynthetic efficiency, thereby producing more photosynthetic products, and finally, positively affects the production and development of the harvestable organs of saffron.

## Figures and Tables

**Table 1:** The abbreviations, definitions, and formula of OJIP parameters in this study.

**Basic Parameters**		
**F_0_**	Minimum fluorescence, when all PSII reaction centers (RCs) are open (O-step of OJIP transient)	F_50µs_
**F_J_**	Fluorescence intensity at the J-step (2 ms) of OJIP	F_2ms_
**F_I_**	Fluorescence intensity at the I-step (30 ms) of OJIP	F_30ms_
**Fluorescence Parameters**		
**F_m_**	Maximum fluorescence, when all PSII RCs are closed (P-step of OJIP transient)	F_1s_
**F_v_**	Variable fluorescence of the dark-adapted leaf	F_m_ − F_0_
**Calculated Parameters**		
**V_J_**	Relative variable fluorescence at time 2 ms (J-step) after the start of the actinic light pulse	(F_J_ − F_0_)/(F_m_ − F_0_)
**V_I_**	Relative variable fluorescence at time 30 ms (I-step) after the start of the actinic light pulse	(F_30ms_ − F_0_)/(F_m_ − F_0_)
**F_v_/F_m_**	The maximal quantum yield of PSII photochemistry	1 − (F_0_/F_m_) = (F_m_ − F_0_)/F_m_= φP_0_
**Quantum Yields and Efficiencies/Probabilities**		
**φ** **_E0_**	The quantum yield of electron transport	[1-(F_0_/F_m_)](1 − V_J_)
**φ** **_D0_**	Quantum yield of energy dissipation	F_0_/F_m_
**φ** **_PAV_**	Average (from time 0 to t_FM_) quantum yield for primary photochemistry	φ_P0_ (1 − V_J_) = φ_P0_ (S_M_/t_FM_)
**TR_0_/ABS**	Specific energy fluxes (per Q_A_ reducing PSII RC)	
**ABS/RC**	The specific energy fluxes per RC for energy absorption	M_0_ (1/V_J_)(1/φ_P0_)
**TR_0_/RC**	Trapped energy flux (leading to Q_A_ reduction) per RC	M_0_ (1/V_J_)
**ET_0_/RC**	Electron transport flux (further than Q_A_^−^) per RC	M_0_ (1/V_J_)(1 − V_J_)
**DI_0_/RC**	Dissipated energy flux	(ABS/RC) − (TR_0_ /RC)
**Performance Indexes (Products of Terms Expressing Partial Potentials at Steps of Energy Bifurcations)**		
**PI_ABS_**	Performance index for the photochemical activity	[(γRC/1 − γRC)(φ_P0_ /1 − φ_P0_)(ψ_E0_/1 − ψ_E0_)]

**Table 2:** Flower properties of saffron plants grown under different ratios of Blue (B) to Red (R) light in the overall light spectral composition, including 100% B, 75% B, 50% B, 40% B, 25% B, 0% B (see the spectrum in Figure 1), with 150 ± 10 μmol m^−2^ s^−1^ PPFD. Means within a column followed by the same letters are not significantly different at *p* ≤ 0.01 according to Duncan’s multiple range test. CV % indicates the percentages of coefficient of variation among the treatments.

Treatment	No. of Flower Per Corm	Flower FW (mg)	Flower DW (mg)	Stigma FW (mg)	Stigma DW (mg)
100% B	2.3 ^a^	622.2 ^a^	112.7 ^a^	54.03 ^a^	16.57 ^a^
75% B	0.98 ^b^	408.3 ^bc^	48.43 ^bc^	44.03 ^b^	9.25 ^b^
50% B	1.3 ^ab^	494.4 ^b^	75.44 ^b^	52.03 ^ab^	13.33 ^ab^
40% B	0.66 ^c^	277.9 ^c^	45.03 ^bc^	35.86 ^c^	5.83 ^c^
25% B	0.77 ^c^	275 ^c^	42.43 ^bc^	24.54 ^c^	4.03 ^c^
0% B	0.66 ^c^	222 ^c^	32.03 ^c^	26.03 ^c^	3.83 ^c^
*p*	<0.0001	<0.0001	0.0027	<0.0001	<0.0001
CV %	20	18.6	15	11.4	15.5

**Table 3:** Leaf and root properties of saffron plants grown under different ratios of Blue (B) to Red (R) light in the overall spectral composition, including 100% B, 75% B, 50% B, 40% B, 25% B, 0% B (see the spectrum in Figure 1), with 150 ± 10 μmol m^−2^ s^−1^ PPFD. Means within a column followed by the same letters are not significantly different at *p* ≤ 0.01 according to Duncan’s multiple range test. CV % indicates the percentages of coefficient of variation among the treatments.

Treatment	No. of Leaves	Leaf FW (mg)	Leaf DW (mg)	Leaf Length (cm)	Root FW (mg)	Root DW (mg)	Root Length (cm)
100% B	15.27 ^d^	4.33 ^c^	0.73 ^b^	46.4 ^d^	4.93 ^a^	0.44 ^a^	3.47 ^a^
75% B	15.53 ^d^	4 ^c^	0.67 ^b^	45 ^d^	3.93 ^b^	0.22 ^c^	3.03 ^ab^
50% B	16.6 ^c^	5.16 ^b^	0.5 ^c^	50 ^c^	5.03 ^a^	0.49 ^a^	3.33 ^a^
40% B	17.3 ^b^	6.5 ^a^	1.66 ^a^	53.03 ^b^	3.5 ^bc^	0.27 ^bc^	2.03 ^b^
25% B	20.77 ^a^	5.5 ^ab^	0.61 ^b^	50.9 ^c^	3.98 ^b^	0.36 ^b^	2.9 ^b^
0% B	18.66 ^ab^	6.33 ^a^	1.03 ^a^	59.77 ^a^	1.83 ^c^	0.14 ^c^	1.77 ^b^
*p*	<0.0001	<0.0001	0.0003	<0.0001	<0.0001	<0.0001	<0.0001
CV %	20.17	17.56	25	16.4	18.43	25	15.5

**Table 4:** Characteristics of saffron plants’ daughter corms grown under different ratios of Blue (B) to Red (R) light in the overall spectral composition, including 100% B, 75% B, 50% B, 40% B, 25% B, 0% B (see the spectrum in Figure 1), with 150 ± 10 μmol m^−2^ s^−1^ PPFD. Means within a column followed by the same letters are not significantly different at *p* ≤ 0.01 according to Duncan’s multiple range test. CV % indicates the percentages of coefficient of variation among the treatments.

Treatment	No. of Daughter Corm Per Mother Corm	Biggest Daughter Corm (g)	Daughter Corm Diameter	Daughter Corm FW (g)	Daughter Corm DW (g)
100% B	2.06 ^c^	6.47 ^a^	23.39 ^a^	10.5 ^a^	5.57 ^a^
75% B	3 ^b^	3.03 ^b^	19.43 ^b^	6 ^b^	2.67 ^c^
50% B	2.88 ^ab^	5.33 ^a^	22.38 ^a^	9.95 ^a^	4.25 ^b^
40% B	3 ^b^	3.07 ^b^	19 ^b^	6.03 ^b^	2.46 ^c^
25% B	3.3 ^b^	2.9 ^bc^	17.86 ^bc^	5.06 ^b^	2.16 ^c^
0% B	4 ^a^	1.97 ^c^	15.66 ^c^	4.03 ^c^	0.83 ^d^
*p*	<0.0001	<0.0001	<0.0001	<0.0001	<0.0001
CV %	11.3	20	6.2	16.7	15.54

## Data Availability

The data presented in this study are available on request from thecorresponding authors. The data are not public.
